# The effect of relative deprivation on aggressive behavior of college students: a moderated mediation model of belief in a just world and moral disengagement

**DOI:** 10.1186/s40359-023-01272-6

**Published:** 2023-09-12

**Authors:** Mengyao Wang, Ming Chen, Zhiyan Chen

**Affiliations:** 1 Institute of Psychology, Department of Psychology, Chinese Academy of Sciences, University of Chinese Academy of Sciences, Beijing, 100049 China; 2 The Second Clinical Medical School, Anhui Medical University, Hefei, 230032 Anhui China

**Keywords:** Relative deprivation, Belief in a just world, Moral disengagement, Aggressive behavior, College students

## Abstract

**Background:**

Previous research found that college students have exhibited a group of concern, and aggressive behavior occurs from time to time in daily life. In order to investigate the effect of relative deprivation on aggressive behavior of college students, this study conducted a moderated mediation model to examine the relationship between relative deprivation, aggressive behavior, belief in a just world, and moral disengagement.

**Methods:**

1169 college students(71.7% female; mean age = 19.41, *SD* = 1.3, range = 17-30years) participated in and completed measures of Relative Deprivation Questionnaire, Belief in a Just World Scale, Moral disengagement Scale and Aggression Questionnaire. The data were analyzed by using a moderated mediation model with SPSS and Process 3.1 macro.

**Results:**

The results revealed that: (1) Relative deprivation significantly positively predicted college students’ aggressive behavior controlling for gender, grade, and age (*B* = 0.45, *p*＜0.001). (2) Belief in a just world played mediating role in relative deprivation and aggressive behavior(indirect effect = 0.04, 95%CI = [0.002,0.007], accounting for 9% of the total effect). (3) The moderated mediator model test showed that moral disengagement regulates the first half of the mediator effect path (*B* = 0.71, *p* < 0.01).

**Conclusion:**

The present study analyzed the moral disengagement mechanisms deeply and shed light on how to decrease the aggressive behavior of college students.

## Introduction

In recent years, frequent incidents of campus violence and conflict have aroused widespread concern among researchers about aggressive behavior. Aggressive behavior refers to destructive behavior that intentionally causes physical or psychological harm to others [1], including physical, aggression, and relational aggression [2]. Research shows that 83.93% of college students exhibit moderate or higher aggression tendencies [3], which seriously affects the psychological health and personality development of the aggression and the victim [4, 5], and even leads to depression, suicide, and other consequences [6]. Therefore, it is of great significance to explore the influencing factors and mechanisms of college students’ aggressive behavior.

### Relative deprivation and aggressive behavior

Previous studies on the mechanisms of college students’ aggressive behavior mainly focused on individual factors such as narcissism and refusal sensitivity [7, 8]; Family factors such as parental marital conflict and childhood adverse experiences [9, 10]; School factors such as school moral atmosphere perception and dormitory social status [11, 12], but few studies have focused on the impact of individual perceived social environment, such as relative deprivation. Relative deprivation refers to a subjective cognitive and emotional experience in which individuals or groups perceive their own disadvantage through horizontal or vertical comparisons with reference groups, and then experience negative emotions such as anger and dissatisfaction [13]. The current negative macro-social environment (for example, the widening gap between the rich and the poor) i tends to make individuals experience a sense of relative deprivation [14]. As a social psychological factor reflecting the macro system, individuals are prone to negative emotional and behavioral reactions after they perceive being deprived, such as anxiety, depression, Internet addiction, aggression and so on [15–18]. According to the general stress theory, in social comparison, college students find themselves at a disadvantage, prone to stress and tension, as well as negative emotions such as anger and dissatisfaction, and then exhibiting aggressive behavior [19]. Empirical research found that college students who perceive a strong sense of deprivation are more likely to exhibit aggressive tendencies [20]. The frustration aggression theory also emphasizes that the occurrence of aggressive behavior is always premised on the setbacks experienced by individuals [21]. When individuals feel that they have lost something that should belong to them, they compensate for their sense of deprivation by aggressing others, and gain instant satisfaction [22].

At present, the relationship between relative deprivation and college students’ aggressive behavior has received some researchers’ attention, but how relative deprivation affects college students’ aggressive behavior remains to be further explored.

### The mediating role of belief in a just world

According to the general aggression model, both individual and environmental factors can have impact on aggressive behavior, including values, attitudes, personality and beliefs [23]. The belief in a just world, as a cognitive factor, enables individuals to believe that the world they live in is fair. In this world, people get what they deserve, and what they get is what they deserve [24]. The belief in a just world can lead people to believe in social order and others, commit to pursuing long-term goals, and promote mental health and personal development [25, 26]. The Emotional Injection Model (AIM) indicates that emotional state can affect individuals’ cognitive judgments, and individuals in positive emotions are more willing to pay attention to social fairness and justice, while negative emotions enhance individuals’ attention to unfair events [27]. Individuals who have long felt deprived tend to view themselves, others, and the world with negative attitudes and perspectives, believing that they cannot control their lives and that the world is not fair and just [28, 29]. Empirical research has also showed that negative emotions can significantly negatively predict belief in a just world [30]. Therefore, it can be inferred that a sense of relative deprivation may lead to a decline in the level of belief in a just world. Moreover, belief in a just world is closely related to aggressive behavior. One of the functions of the belief in a just world is to make people more identify with the principles of justice and be willing to follow them [31]. Individuals with higher levels of belief in a just world believe that they will be treated fairly, tend to adopt adaptive ways to cope with stress and conflict, have a high level of life satisfaction, and exhibit more altruistic behavior [32]. Conversely, it can trigger negative emotions and aggressive behavior. Empirical research also shows that belief in a just world is positively correlated with forgiveness behavior, depression and anxiety [33][30, 34, 35] and can also negatively predict problematic behavior [36]. In addition, studies have also confirmed that fair belief in a just world play a mediating role in the relationship between negative factors and problematic behavior. For example, psychological abuse in childhood can indirectly affect college students’ online bullying behavior through belief in a just world [32], and belief in a just world play a mediating role in negative life events and online aggression behavior [37]. Based on this, this study proposed hypothesis 1: Belief in a just world may mediate the relationship between relative deprivation and aggressive behavior.

### The moderating role of moral disengagement

Individual environment interaction model believes that the interaction between individuals and environmental factors jointly affects individual development [38, 39]. Based on this model, relative deprivation is a psychological variable that depends on the social environment, while moral disengagement is a personal trait [40, 41]. The two are not only considered as core factors that affect the individual development of adolescents [22, 42], but their interaction has also received great attention [43]. Moral disengagement refers to the separation of an individual’s internal moral standards from external behavior, reinterpretation of one’s behavior to rationalize it, minimizing guilt and responsibility for the behavior, in order to avoid self-punishment and maintain a moral image [44]. Personal morality is formed during the process of socialization, which makes individuals more conform to social norms in terms of cognition, emotion, and behavior. Under the influence of social requirements, individuals are prone to generate perceptions and beliefs such as “good and evil will eventually be rewarded”, namely, the belief in a just world. Previous studies have shown that moral personality and ability positively predict belief in a just world [45]. Moral disengagement, to a certain extent, reflects the level of individual moral quality [46]. It helps individuals whitewash their immoral thoughts and behaviors and calmly violate social order. Frequent use of moral disengagement mechanisms will gradually establish chaotic moral norms and distorted values, and internalize them into personal characteristics. Empirical research has also found that there is a significant negative correlation between moral disengagement and belief in a just world [47, 48]. The risk enhancement model indicates that the role of a single risk factor is relatively limited. When two risk factors act together on an individual, the impact generated is far greater than the simple sum of the two risk factors, which may bring more serious consequences. Therefore, this study speculates that when college students with high moral disengagement feel deprived, they are more likely to use one or more moral disengagement mechanisms to get rid of their own responsibilities, and lower moral standards with peace of mind, finally reduce their belief in the justice of the world. Hence, this study proposes hypothesis 2: Moral disengagement plays a moderated role in the first half of the mediating pathway.

**Fig. 1:**
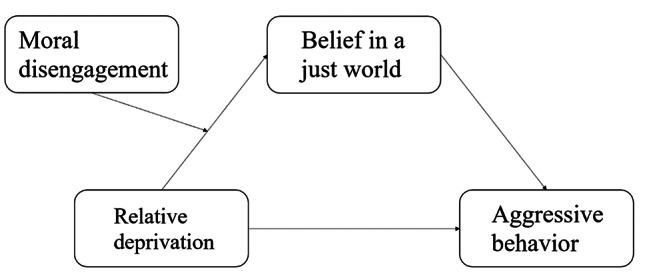
A model of the relationship between relative deprivation, belief in a just world, aggressive behavior and moral disengagement

In summary, based on the integration of the general aggression model and the individual environment interaction model, this study constructed a moderated mediation model (see Fig. 1). This study aims to answer the question of “how” and “under what circumstances” relative deprivation affects college students’ aggressive behavior, and provide theoretical guidance for the prevention and intervention of college students’ aggressive behavior.

## Materials and methods

### Participants

Using a convenient sampling method, 1259 college students in Zhejiang Province were selected for testing, and 1169 (92.9%) valid questionnaires were collected. The age distribution of the subjects was 17–30 years old, with an average age of 19.41 ± 1.3. Among them 330 were male and 839 were female. There are 606 only children and 563 non only children. This study was approved by the local Ethics. All participants provided written informed consent. All procedures were in accordance with the ethical standards of the responsible committee on human experimentation and with the Helsinki Declaration.

### Measures

#### Relative deprivation questionnaire

Referring to Ma Ai’s (2012) [49], we measured relative deprivation using four topics. For example, “I always feel like someone else has something that belongs to me.“. The scale is scored at 6 levels, with 1 indicating “very disapproval” and 6 indicating “very approval”. The result analysis uses an average score. The higher the score, the higher the individual’s relative sense of deprivation. In this study, Cronbach’s α is 0.72.

#### Belief in a Just World Scale

The Belief in a Just World Scale [36], developed by Dalbert (1999) and revised by Su et al. (2011), has 13 items, including two subscales: individual just world beliefs and general just world beliefs. A score of 6 levels is used, with 1 indicating “very disapproval” and 6 indicating “very approval”. The result analysis uses an average score. The higher the score, the stronger their belief in a just world. Cronbach’s α of the scale in this study is 0.93.

#### Moral disengagement Scale

Chinese version of the Moral disengagement Questionnaire revised by Wang Xingchao and Yang Jiping (2010) [49] was used. There are 26 items in total, including eight aspects: moral defense, euphemistic labeling, favorable comparison, responsibility transfer, responsibility dispersion, distorted results, dehumanization, and blame attribution. A score of 5 levels is used, with 1 being “completely disagree” and 5 being “fully agree”. The result analysis uses an average score. The higher the score, the higher the level of moral disengagement. Cronbach’s α of the scale in this study is 0.92.

#### Aggression questionnaire

Aggression questionnaire developed by Buss and Perry (1992) [50] was used. There are 29 items in total, including physical aggression, verbal aggression, anger, and hostility. Using a 5-level scoring system, 1 is “completely non compliant” and 5 is “fully compliant”, with 9 and 16 questions scored in reverse. The result analysis uses an average score. The higher the score, the higher the individual’s aggression level. Cronbach’s α of the scale in this study is 0.88.

### Data analysis method

SPSS 23.0 was used for descriptive statistics, correlation analysis, and regression analysis. Process 3.1 macro was used for mediating and moderated effects analysis.

## Results

### Inspection and control of common method bias

Since data were collected using a self-reported method, the results may be affected by common method bias. In addition to using anonymous testing, reverse scoring, this study also used Harman’s single factor test method to conduct non rotated principal component analysis of the measured items. The data generated a total of 14 common factors with a characteristic value greater than 1, and the interpretation amount of the first factor was only 17.4%, less than 40%. No serious common method bias was observed.

### Descriptive statistics and correlation analysis

Table 1 lists the mean, standard deviation and correlation matrix for each variable. The results showed that aggressive behavior was significantly negatively correlated with belief in a just world(*r* = -0.26, *p*＜0.001); relative deprivation was significantly positively correlated with moral disengagement (*r* = 0.24, *p*＜0.001) and aggressive behavior (*r* = 0.44, *p*＜0.001); belief in a just world significantly negatively correlated with moral disengagement (*r* = -0.13, *p*＜0.001).

**Table 1:** Descriptive statistics and correlation analysis results of each variable

	*M*	*SD*	1	2	3	4
1 Relative deprivation	2.65	0.75	1			
2 Moral disengagement	2.31	0.61	0.24^***^	1		
3 Belief in a just world	3.98	0.71	-0.28^***^	-0.13^***^	1	
4 Aggressive behavior	2.58	0.50	0.44^***^	0.30^***^	-0.26^***^	1

*p < 0.05 ,**p < 0.01, ***p < 0.001, the same below

### Moderated mediation model test

According to the suggestions of Wen Zhonglin and Ye Baojuan [50–52], all variables were standardized, and then adjusted mediating effect analysis was conducted under the control of gender and age. All analysis processes were conducted using the SPSS macro PROCESS 3.1. The bias corrected percentile Bootstrap method was used for testing, and 5000 repeated samples were taken to calculate a 95% confidence interval. The specific results are shown in Table 2 (for simplicity, referring to the practice of Wei Hua [53] et al., the regression coefficients for gender, age, and whether an only child or not are not listed in the table).

**Table 2:** Mediation model test of belief in a just world

Variables	Belief in a just world (Step 1)				Aggressive behavior (Step 2)			
	*B*	*SE*	*t*		*B*	*SE*	*t*	
Relative deprivation	-0.28	0.03	-9.90^***^		0.41	0.27	14.98^***^	
Belief in a just world					-0.15	0.03	-5.38^***^	
*R* ^*2*^	0.08				0.22			
*F*	25.74^***^				65.52^***^			

Note: All variables in the model are brought into the regression equation after standardization,the same below

The first step was to test the simple mediation model (model 4), the results were shown in Table 2. Regression analysis showed that relative deprivation had a significant positive predictive effect on aggressive behavior (*B* = 0.45, *p*＜0.001); After incorporating belief in a just world into the regression equation, relative deprivation still had a significant positive predictive effect on aggressive behavior (*B* = 0.41, *p* < 0.001), relative deprivation significantly negatively predicted belief in a just world (*B*=- 0.28, *p* < 0.001), belief in a just world significantly negatively predicted aggressive behavior (*B*=- 0.15, *p*＜0.001). ab = 0.04, Boot *SE* = 0.01, 95% confidence interval was [0.02, 0.07], excluding 0. It indicated that belief in a just world played a partial mediating role in the impact of relative deprivation on aggressive behavior. The proportion of intermediary effect to total effect was 9%.

**Fig. 2:**
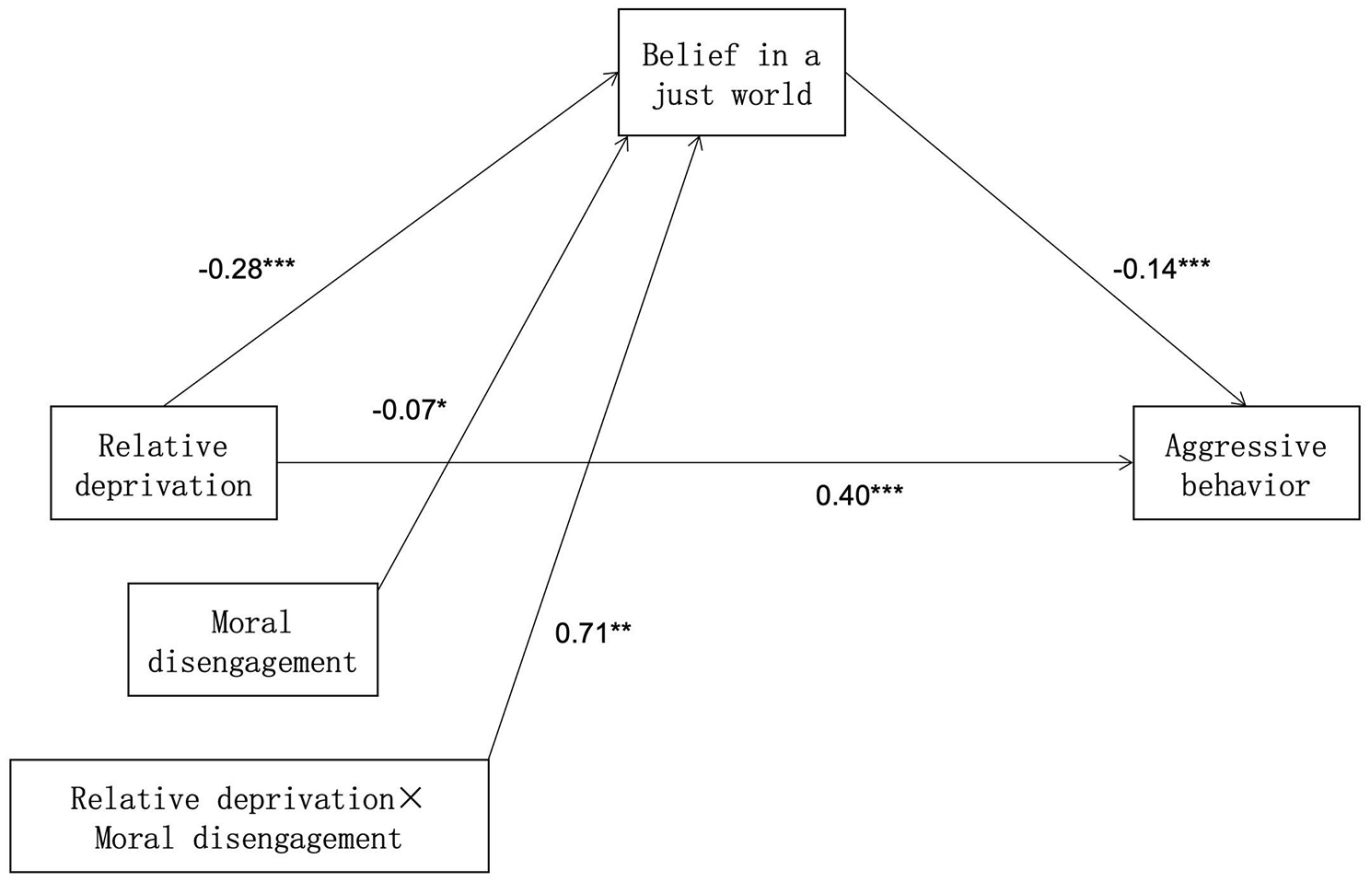
The summary figure for model 7 in this study

The second step was to test the moderated mediation model (model 7). The results were shown in Table 3; Fig. 2. Regression analysis showed that relative deprivation negatively predicted belief in a just world (*β*=- 0.27, *p* < 0.001), moral extrapolation negatively predicted the belief in a just world (*β*=- 0.07, *p* < 0.05), and the interaction between relative deprivation and belief in a just world had a significant predictive effect on belief in a just world (*β* = 0.71, *p* < 0.01), with a 95% confidence interval of [0.03, 0.11], excluding 0. This result indicated that moral disengagement played a regulatory role in the first half of the intermediary path of “relative deprivation → belief in a just world → aggressive behavior”.

**Table 3:** Bias of mediating moderating effect of relative deprivation on aggressive behavior

Variables	Belief in a just world (Step 1)				Aggressive behavior (Step 1)				Belief in a just world (Step 2)		
	*B*	*SE*	*t*		*B*	*SE*	*t*		*B*	*SE*	*t*
Relative deprivation	-0.28	0.03	-10.00^***^		0.40	0.27	14.94^***^		-0.27	0.03	-9.26^***^
Belief in a just world					-0.14	0.03	-5.34^***^				
Moral disengagement									-0.07	0.29	-2.42^*^
Relative deprivation×Moral disengagement									0.71	0.02	3.31^**^
*R* ^*2*^	0.08				0.22				0.09		
*F*	100.17^***^				160.98^***^				39.10^***^		

**Fig. 3:**
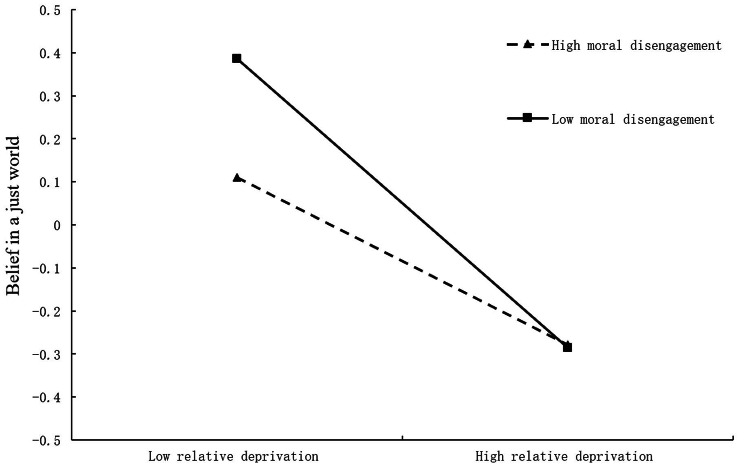
The interaction between moral disengagement and relative deprivation on belief in a just world

In order to reveal how moral reasoning moderated the relationship between relative deprivation and belief in a just world, a simple slope test was conducted. The simple effect analysis diagram was drawn (Fig. 3) based on the value of moral reasoning by grouping high and low (plus or minus a standard deviation). The results showed that when the level of individual moral disengagement was low (-1*SD*), the positive predictive effect of relative deprivation on belief in a just world was significant (simple slope =-0.34, *SE* = 0.04, *p* < 0.001), with a 95% confidence interval of [− 0.41, − 0.26]. When the level of individual moral disengagement was high (+ 1*SD*), the positive predictive effect of relative deprivation on belief in a just world was smaller (simple slope =-0.18, *SE* = 0.04, *p* < 0.001), with a 95% confidence interval of [− 0.27, − 0.12]. In short, as the level of moral disengagement increased, the predictive effect of relative deprivation on college students’ belief in a just world decreased.

## Discussion

This study examined the impact of relative deprivation on college students’ aggressive behavior and its underlying mechanisms. The results showed that relative deprivation could positively predict college students’ aggressive behavior, which was consistent with previous research conclusions [22, 54–56]. It can be seen that relative deprivation, a social environmental factor, is an important predictor of aggressive behavior. When college students experience frustration compared to others, they may experience resentment and jealousy, which can lead to aggressive behavior. In addition, it is also necessary to further explore how relative deprivation affects college students’ aggressive behavior and under what circumstances does it affect aggressive behavior. Results of the study have certain implications for deepening the relevant research on the impact mechanisms of college students’ aggressive behavior, as well as the prevention and intervention of college students’ aggressive behavior.

This study showed that belief in a just world played a mediating role between relative deprivation and college students’ aggressive behavior. That was, the effect of relative deprivation on college students’ aggressive behavior was achieved through direct prediction of their aggressive behavior and the mediating role of belief in a just world. Hypothesis 1 was supported and consistent with previous research [57]. An individual’s belief in a just world can be influenced by emotions. College students who experience a sense of relative deprivation may perceive themselves as inferior to others and are prone to emotions such as resentment and jealousy. Repeated stimulation of individuals with negative self-cognition and negative emotions can undermine their cognitive schema [58], making them question or even deny the notion that “good is rewarded with good, and evil is rewarded with evil.“. If the unfair information that individuals are exposed to accumulates to a certain extent, their belief in a just world will be weakened [59]. Previous studies have pointed out that belief in a just world is beneficial for individuals to reduce negative emotions, improve problem-solving abilities, and conform their behavior to social norms [31, 60]. Individuals with lower belief in a just world always believe that they have not been treated fairly, lack trust in others and the world when faced with pressure, and have more intense anger [61]. Accordingly, they tend to adopt more non adaptive behaviors to “maintain fairness and justice” [62].

This study also found that moral disengagement moderated the first half of the path that relative deprivation affects college students’ aggressive behavior through belief in a just world. However, the results showed that rather than increasing, high moral disengagement delayed the negative effect of relative deprivation on college students’ beliefs about a just world, which contradicts hypothesis 2. It was also not conform to the risk enhancement model. The following explanation may help us understand this result: Previous studies have shown that only individuals with low levels of moral reasoning are susceptible to negative situational factors [63]. In other words, college students with lower levels of moral disengagement have a basic moral outlook and are able to face life events more rationally. At this time, relative deprivation has a strong predictive effect on the belief in a just world. On the contrary, when the level of moral disengagement of college students reaches a certain level, the moral self-regulation mechanism has failed [64], and their moral belief level rapidly decreases. Under such circumstance, individuals can hardly believe in the fairness and justice of the world, whether they feel deprived or not. At this time, the predictive effect of relative deprivation on the belief in a just world is relatively weakened. This result suggested that we should make college students understand more about the mechanisms of moral disengagement and avoid internalizing moral disengagement into individual traits.

### Research significance and deficiencies

This study explored the impact of relative deprivation on aggressive behavior through a moderated mediation model. Theoretically, it helps to understand how relative deprivation affects college students’ aggressive behavior, and when the impact is more significant. At the same time, it provides some empirical evidence for general aggression model, individual environment interaction theory. After reviewing literatures, it is found that most of the previous studies have explored the impact of the interaction of personal traits (trait anger, psychotic traits, trait self-control, empathy, etc.) and moral disengagement on individual psychology [63]. Their findings showed a “risk enhancement” pattern, which indicated that the interaction of certain negative personal traits and moral disengagement would bring more serious consequences to individuals [65]. However, this study is the first to examine the influence of the interaction between an individual variable (relative sense of deprivation) and moral disengagement that depends on environmental factors, and it presents different results from previous studies, namely: the relationship between individuals with high moral disengagement and relative sense of deprivation, the interaction not only cannot play the role of “adding insult to injury”, but delays the impact of relative deprivation on personal psychological factors (belief in a just world)[66]. In other words, this study found that the trait of moral disengagement itself has a strong predictive effect on the individual’s psychological development, and individuals with a high level of moral disengagement are not easily affected by environmental factors [67].

At the practical level, the findings of this study remind us: First, we should pay attention to the influence of moral cognitive factors in the prevention and treatment of college students’ aggressive behavior. School psychologists and educators should pay extra attention to students with high levels of moral disengagement, and provide psychological counseling and educational guidance to these teenagers in terms of moral cognition. Second, unlike the absolute deprivation of the objective state, the sense of relative deprivation is a subjective state, can be effectively intervened and improved in a short period of time through cognitive training. Finally, pay attention to the cultivation and guidance of college students’ belief in a just world, establish a reasonable belief in a just world, and use positive encouragement to reduce their aggressive behavior.

In addition, this study also has some limitations. Firstly, the cross-sectional design cannot reveal the dynamic changes of relative deprivation in aggressive behavior. Future research can combine longitudinal design to examine the relationship between them. Secondly, the use of self-reporting may increase social expectation effects. In the future, it may be considered to obtain data through multiple channels (such as teacher evaluation, peer evaluation). Thirdly, due to the large gap in the gender ratio in the sampled schools, the gender ratio in the study sample is not uniform enough, which may limit the popularization of this study. Fourthly, all participants were from one university in a local city. The sampling range is a bit narrow and the representativeness of the sample is not high. Future research is needed with large sample from different cities to test our findings. Finally, whether there are other underlying mechanisms in the effects of relative deprivation on college students’ aggression behavior besides moral disengagement remains to be supplemented by future research.

## Conclusion

This study showed that relative deprivation is associated with increased aggressive behavior through decreasing belief in a just world, and this effect was particularly pronounced among less moral disengagement groups. The findings highlighted the importance to develop prevention and intervention regarding relative deprivation, as a way to prevent aggression among college students.

## Data Availability

The data that support the findings of this study are available on request from the corresponding author.
